# Impact of China’s National Volume-Based Procurement policy exclusively for insulin on the volume, expenditure and price: an interrupted time series analysis in Guangdong Province

**DOI:** 10.3389/fpubh.2025.1659721

**Published:** 2025-09-08

**Authors:** Changsong Jiang, Manling Nie, Yun Lu, Ting Jiang, Dan Guo, Peng Qi, Na Li, Feng Chang

**Affiliations:** ^1^School of International Pharmaceutical Business, China Pharmaceutical University, Nanjing, China; ^2^National Institute of Healthcare Security, Capital Medical University, Beijing, China; ^3^HEOA Group, West China School of Public Health and West China Fourth Hospital, Sichuan University, Chengdu, China

**Keywords:** National Volume-Based Procurement, insulin, value-based medicine, availability, utilization pattern, interrupted time series analysis

## Abstract

**Objective:**

This study aims to evaluate the effect of China’s National Volume-Based Procurement (NVBP) policy exclusively for insulin by analyzing the trend in volume, expenditure, and price before and after NVBP policy.

**Methods:**

Taking Guangdong Province, China as an example, descriptive statistics and interrupted time series analysis were used to quantitatively measure the immediate and long-term effect of the NVBP policy on insulin volume, expenditure and price. In terms of volume, subgroup analysis is further conducted based on different generations and enterprise ownership to examine the impact of NVBP on the insulin utilization pattern.

**Results:**

Following the implementation of the NVBP policy, monthly insulin procurement volume increased significantly from 7.69 million to 9.37 million defined daily doses (DDDs), while monthly expenditure decreased from CNY 86.64 million to CNY 52.55 million, accompanied by a reduction in defined daily dose cost (DDDc) from CNY 11.24 to CNY 5.57. Interrupted time series analysis (ITSA) confirmed these trends, showing an immediate post-intervention increase of 1.547 million DDDs (*p* < 0.001), expenditure reduction of CNY 42.57 million (*p* < 0.001), and DDDc decrease of CNY 5.427 (*p* < 0.001) in instantaneous level. Subgroup analysis demonstrated divergent trends between insulin generations, with non-significant decrease in second-generation insulin and increase in third-generation insulin procurement in long-term trend. Notably, domestic insulin showed a significant increase in procurement volume (*p* < 0.05), while imported insulin exhibited a non-significant declining trend.

**Conclusion:**

These findings demonstrate that the NVBP policy significantly reduced insulin expenditure while improving treatment accessibility and affordability for insulin. The policy effectively promoted therapeutic upgrading from human insulin to insulin analogue and optimized medication regimen. Notably, it stimulated domestic insulin market development through substitution effect. This multi-dimensional improvement exemplifies the principles of value-based healthcare delivery.

## 1 Introduction

According to the International Diabetes Federation (IDF) *Global Diabetes Atlas* (11th edition, 2025), there are 148 million adults aged 20–79 with diabetes in China (1), the highest number globally, accounting for approximately a quarter of the world’s diabetic patients. Diabetes often refers to the “non-lethal cancer,” and is the fundamental progenitor of multi-organ complications (2–4). Patients with diabetes need to inject insulin lifelong or take other glucose-lowering drugs, contributing to substantial economic costs. The inability to access affordable insulin remains a critical barrier to effective treatment, leading to unnecessary complications and premature mortality (5).

To alleviate the financial burden on patients, the National Healthcare Security Administration (NHSA) launched a nationwide centralized volume-based procurement policy for insulin in November 2021, which is the only procurement for biologics in China at the national level. The procurement involves second-generation human insulin and third-generation insulin analogue. Insulin analogue as upgraded product of human insulin, is generally more expensive but more stable in controlling blood glucose and significantly reducing the risk of hypoglycemia (6). The centralized procurement targeted commonly used insulin types in clinical practice to maintain stability in clinical medication use. The insulin was categorized into six bidding groups in process of procurement, including mealtime human insulin, basal human insulin, premixed human insulin, rapid-acting analogue (aspart, lispro, glulisine), long-acting basal analogue (glargine, detemir, degludec) and premixed analogue (protamine lispro and lispro, protamine aspart and aspart). Different from chemical drugs, insulin production is more complex, with longer production cycle, higher cost, and limited capacity expansion in the short term (7). Therefore, centralized procurement for insulin differs from that of chemical drugs in terms of rule design, competition mechanism, and supply of bid-winning companies. Medical institutions can report their demand of insulin by brand name before procurement, whereas chemical drugs are reported by their generic name. In order to narrow the price gap, the unit price of insulin must not exceed 1.3 times the lowest unit price within the same bidding group to be selected, whereas for chemical drugs, this threshold is 1.8 times. Insulin bids can be selected if the price is below 60% of the highest valid bid, while chemical drugs require a bid below 50%. In summary, insulin competition is milder, and price reduction is smaller. In terms of supply, multiple selected enterprises provide insulin, allowing medical institutions to choose freely, ensuring market supply and respecting clinical choice to better guarantee patient accessibility. In contrast, for chemical drugs, a single selected enterprise supplies most of the volume within a province.

The NVBP for insulin involved 14,000 medical institutions nationwide, with a total demand volume of 215 million shots in the first year (8). Of this, human insulin accounted for 42%, while insulin analogue accounted for 58%. Domestic insulin made up 32% of the demand, and imported insulin represented 68%. Major insulin manufacturers such as Novo Nordisk, Eli Lilly, Sanofi, and seven domestic companies were winning bidders, the average price reduction was 48% (9). The procurement result was implemented starting in May 2022 across provinces, with a two-year procurement cycle.

The NVBP policy is of great significance for reducing patient financial burden, saving medical expenditure, and optimizing the allocation of medical resource (10–12). It is also a key driver for achieving value-based medicine, which focuses on patient health outcome, aiming to achieve healthcare system sustainability by optimizing resource allocation, controlling costs, and improving service quality (13). The NVBP policy leverages volume-based bargaining and market access to synergy government and market forces, promoting efficient allocation of medical resource. And it helps to improve health outcome of some patients by enhancing patients’ accessibility to drugs after price reduction. While some academic studies have empirically analyzed the impact of NVBP for chemical drugs (14–18), there are few research on the effect of NVBP for biologics, only Yuan et al.’s study has introduced the procurement mechanism of the NVBP for insulin and the impact on affordability of patients (7).

This study aims to evaluate the impact of the NVBP policy exclusively in three key aspects: (1) Whether the policy effectively improves the accessibility and affordability of insulin, saves insulin expenditure, and enhances the sustainability of medical expenditure. (2) Under the context of value-based medicine, whether the policy helps to optimize the drug utilization pattern by increasing the share of insulin analogue and promoting the use of high-quality insulin analogue among more patients. (3) Whether the policy reduces the dependence on imported insulin, encourages the substitution of domestic insulin, and mitigates supply chain risks.

Guangdong Province, located in southern China, with a total population of 127 million, representing 10% of China’s population, had a per capita GDP of CNY 106,985 and a per capita disposable income of CNY 49,327 in 2023 (19). Guangdong ranks among the top regions in China in terms of economic development level, healthcare coverage, and proportion of young people, playing a significant role nationally. However, there is a significant income disparity across different regions of the province, with the Pearl River Delta (PRD) region far surpassing the northwestern part. In 2023, the province had 62,819 medical institutions, with 4.95 hospital beds and 92.02 healthcare personnel per 1,000 people (19). Despite the overall abundance of healthcare resources, the distribution is uneven—nearly 70% of medical institutions are concentrated in the PRD, making it a microcosm of healthcare inequality in China properly. There are over 8 million diagnosed diabetes cases in Guangdong (20), ranking among the highest prevalence regions nationally. This study used insulin procurement data from Guangdong Province for quantitative descriptive analysis and policy intervention modeling. The findings provide evidence for evaluating the impact of the NVBP policy on insulin accessibility and affordability.

## 2 Methods

### 2.1 Data sources

The data were obtained from the Guangdong provincial centralized drug procurement and trading platform (21), through which all pharmaceutical procurement and transactions by medical institutions and pharmaceutical enterprises across the province are conducted. Monthly insulin procurement transaction records from May 2020 to June 2024 were adopted in this study, and the procurement transaction records including key variables such as procurement time, drug name, brand name, dosage form, package specification, manufacturer, procurement volume, and procurement expenditure. The implementation time of the NVBP policy for insulin in Guangdong Province was May 31, 2022, with a procurement cycle of 2 years. This study designates June 2022 as the policy intervention point and conducts analysis using procurement data from the 25 months preceding (from May 2020 to May 2022) and the 25 months following the intervention (from June 2022 to June 2024).

### 2.2 Statistical analysis

Descriptive statistics were conducted on the procurement volume, expenditure, and defined daily dose cost of insulin before and after the NVBP policy, as well as for different subgroups. The volume was measured as Defined Daily Doses (DDDs), which is the ratio of the total consumption of the drug to its Defined Daily Dose (DDD).


$$\text{DDDs}=\frac{\text{unit strength}\times \text{pack size}\times \text{procurement amount}}{\mathrm{DDD}}$$

The DDD, representing the average daily dose used in the principal indication for adults, was set at 40 IU for all insulin based on the WHO ATC/DDD index (22). Expenditure was represented by procurement costs, while the price was measured by the Defined Daily Dose cost (DDDc), calculated as the ratio of procurement costs to DDDs.


$$\text{DDDc}=\frac{\text{procurement costs}}{\text{DDDs}}$$

Interrupted time series analysis is considered one of the most robust quasi-experimental methods for evaluating the longitudinal impact of policy interventions (23). The ITS was employed using volume, expenditure, and DDDc as outcome variables to assess the immediate and long-term effect of the NVBP on insulin usage and costs. A single-group ITS model was constructed as follows (24, 25):


$$Y_{t}=\beta_{0}+\beta_{1}T_{t}+\beta_{2}X_{t}+\beta_{3}P_{t}X_{t}+\beta_{4}D_{t}+\in_{t}$$

Where, Y_t_ indicates the measured outcome variable in month t. T_t_ denotes the time series number, which corresponds to each observation point. The number “1” is assigned to April 2020, and “50” is assigned to June 2024. X_t_ is a dummy variable indicating the intervention, where “0” represents the period before NVBP and “1” represents the period after the implementation of NVBP. P_t_ is the time series after intervention, which value is “0” before intervention and is denoted in sequence according to the monthly order after intervention. Besides, a dummy variable D_t_ is introduced to control the influence of the Chinese Spring Festival holiday on the transaction between medical institutions and pharmaceutical enterprises (14, 26). During the Spring Festival holiday, which lasts for about one-third of the month in February, insulin transactions between medical institutions and pharmaceutical enterprises tend to decrease significantly. Both the procurement volume and the expenditure show a marked decline in February. Therefore, D_t_ is set to 1 for February and set to 0 for other months.

In this model, *β*₀ represents the initial level of the outcome variables at the start of the study. *β*₁ represents the slope before the intervention. *β*₂ represents the level change of outcome variables at the intervention moment. β₃ represents the slope change after the intervention. β₄ represents the level change of outcome variable in abnormal months.

The Durbin-Watson test was employed to assess autocorrelation (27). Residual plots were visually inspected to identify heteroscedasticity (28, 29). Models were weighted using the inverse of squared residuals when heteroscedasticity occurred. The R4.4.1 software was used to perform statistical analysis for the study.

## 3 Results

### 3.1 Changes in insulin procurement volume, expenditure, and DDDc following NVBP implementation

The volumes of insulins in Guangdong Province increased after NVBP, while the expenditure and DDDc significantly decreased compared with those before volume-based procurement. The results are shown in Table 1.

**Table 1:** Insulin volume and expenditure in Guangdong Province, from May 2020 to June 2024.

Categories	Before the NVBP (2020.05–2022.05)			After the NVBP (2022.06–2024.06)		
	Volume/million DDDs	Expenditure/million CNY	DDDc/CNY	Volume/million DDDs	Expenditure/million CNY	DDDc/CNY
Generations						
Human insulin	52.47	267.95	5.11	58.41	191.91	3.29
Insulin analogue	139.92	1897.95	13.53	177.39	1121.75	6.32
Enterprises						
Domestic insulin	61.27	487.76	7.94	91.67	387.12	4.23
Imported insulin	131.13	1678.14	12.76	144.13	926.55	6.42
Bidding groups						
Basal human	0.83	4.81	5.85	1.34	5.20	3.88
Mealtime human	21.00	75.78	3.61	27.48	76.39	2.78
Premixed human	30.65	187.36	6.12	29.60	110.32	3.73
Long-acting basal analogue	46.10	1027.74	22.29	66.48	596.93	8.98
Rapid-acting analogue	37.72	355.94	9.44	52.75	260.83	4.95
Premixed analogue	56.10	514.27	9.16	58.15	263.98	4.53
Total	192.40	2165.90	11.24	235.80	1313.66	5.57

The volume and expenditure refer to the cumulative procurement volume and expenditure of various insulin categories, respectively. DDDc represents the arithmetic mean of monthly DDDc for various insulin categories. NVBP, national volume-based procurement; DDDs, defined daily doses; DDDc, defined daily dose cost.

(1) The average monthly volume of insulins was 7.69 million DDDs before NVBP, which increased to 9.37 million DDDs after centralized procurement. The total procurement volume of insulins rose from 192.40 million DDDs to 235.80 million DDDs. (2) The average monthly expenditure of insulins was CNY 86.64 million before centralized procurement, decreasing to CNY 52.55 million after centralized procurement. The total procurement expenditure decreased from CNY 2165.90 million to CNY 1313.66 million, which reduced by 39.35%. (3) The DDDc of insulins decreased from CNY 11.24 to CNY 5.57 after centralized procurement, representing a year-on-year decrease of approximately 50%.

Subgroup analyses further differentiated these effects across insulin generations (human insulins vs. insulin analogues), manufacturer types (domestic insulins vs. imported insulins) and bidding groups, with detailed results presented in Table 1.

### 3.2 Insulin procurement volume, expenditure, and DDDc across different generations

The average monthly volume of human insulins increased from 2.10 million DDDs to 2.34 million DDDs after the implementation of NVBP, while the average monthly volume of insulin analogues rose from 5.60 million DDDs to 7.10 million DDDs. Besides, the proportion of human insulins procurement decreased from 27.27% before the procurement to 24.77%, while the proportion of insulin analogues increased from 72.73 to 75.23%. The average monthly expenditure for human insulins decreased from CNY 10.71 million to CNY 7.68 million, and the total expenditure decreased from CNY 267.95 million to CNY 191.91 million; The average monthly expenditure for insulin analogues decreased from CNY 75.92 million to CNY 44.87 million, and the total procurement expenditure decreased from CNY 1,897.95 million to CNY 1,121.75 million. The DDDc difference between the two generations of insulin before and after the NVBP were CNY 8.42 and CNY 3.03, respectively, which have been narrowing following the NVBP policy.

### 3.3 Insulin procurement volume, expenditure, and DDDc across different enterprises

The volume for both domestic insulins and imported insulins increased following the NVBP policy. The average monthly volume of domestic insulins rose from 2.45 million DDDs to 3.67 million DDDs (a 49.62% increase), while the average monthly volumes of imported insulin increased from 5.24 million DDDs to 5.76 million DDDs (a 9.92% increase). The market share of domestic insulin grew from 31.84 to 38.88%.

Procurement expenditure declined for both insulins, with a sharper reduction for imported insulins (44.79% decline) than for domestic insulins (20.63% decline). Expenditure for domestic insulins decreased from average monthly CNY 19.51 million to average monthly CNY 15.48 million, while imported insulins expenditure fell from average monthly CNY 67.12 million to average monthly CNY 37.06 million.

The DDDc difference between the two market share distribution insulins narrowed significantly, decreasing from CNY 4.82 to CNY 2.19.

### 3.4 Insulin procurement volume, expenditure, and DDDc across different bidding groups

Overall, except for the premixed human insulin, procurement volume increased for the remaining groups, with the basal human insulin showing the highest growth at 61.04%. The procurement expenditure of basal and mealtime human insulin increased post-policy, while other groups decreased. The DDDc of six bidding groups decreased, with the long-acting basal insulin analogue experiencing the most substantial reduction—from 22.29 CNY to 8.98 CNY.

The volume of basal human insulin surged from 0.83 million DDDs to 1.34 million DDDs (a 61.4% increase), while its expenditure increased by 8.1%. The volume of mealtime human insulin rose by 30.9%, with a small increase in its expenditure. However, premixed human insulin was the only group to experience a decrease in volume, with expenditure reducing from CNY 187.36 million to CNY 110.32 million. Among insulin analogues, the most notable divergence was observed in the long-acting basal analogue, whose volume increased by 44.2%, expenditure decreased by 41.9%, and DDDc dropped by 59.7%. The reduction in DDDc of long-acting basal analogues is the most significant, but the DDDc post-policy of long-acting basal analogue still exceeds that of other groups. The volume of rapid-acting analogue increased from 37.72 million DDDs to 52.75 million DDDs (a 39.8% increase), with a 26.7% reduction in expenditure. The price of premixed analogue halved (DDDc dropped by 50.5%), with the volume stagnated with a modest 3.7% increase.

### 3.5 ITS analysis for insulin procurement volume, expenditure, and DDDc

**Figure 1:**
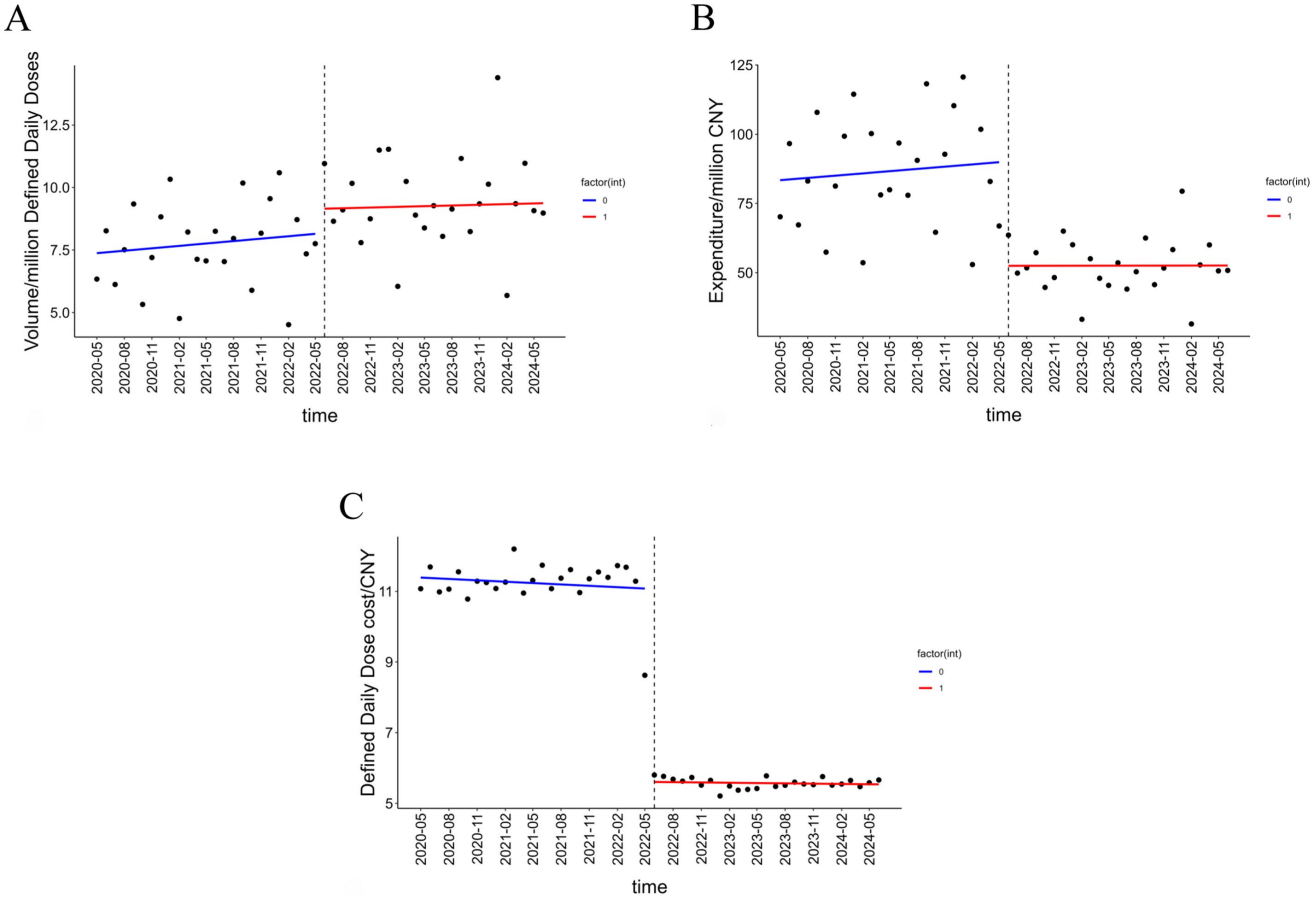
Trends in insulin volume, procurement expenditure, and DDDc. **(A)** The volume trend of insulin before and after the NVBP policy. **(B)** The expenditure trend of insulin before and after the NVBP policy. **(C)** The price trend of insulin before and after the NVBP policy.

The results of the ITS analysis for all insulin categories are presented in Table 2. A statistically significant instantaneous level increase of 1.547 million DDDs (*p* < 0.001) was observed in volume. Following the intervention, the procurement volume showed an upward trend change, but this change was not statistically significant (see Figure 1A).

**Table 2:** Results of ITS analysis for insulin volume, expenditure, and DDDc.

Insulin categories	Baseline level	Baseline trend	Level change	Trend change
Volume/million DDDs				
Overall	7.32^***^	0.007 (0.612)	1.547^***^	0.008 (0.629)
Human insulin	2.039^***^	−0.002 (0.616)	0.439^***^	−0.009 (0.080)
Insulin analogue	5.299^***^	0.009 (0.292)	1.13^***^	0.017 (0.149)
Domestic insulin	2.296^***^	0.003 (0.392)	0.978^***^	0.016^**^
Imported insulin	5.04^***^	0.006 (0.477)	0.579^***^	−0.009(0.346)
Expenditures/million CNY				
Overall	81.18^***^	0.71(0.078)	−42.57^***^	−0.722(0.175)
DDDc/CNY				
Overall	11.172^***^	0.014(0.333)	−5.427^***^	−0.042^*^

All models exhibited statistically significant results (*p* < 0.05) with no evidence of autocorrelation. *p* values corresponding to coefficients are reported in parentheses; *p < 0.05, **p < 0.01, and ***p < 0.001. ITS, interrupted time series; DDDs, defined daily doses; DDDc, defined daily dose cost.

A statistically significant instantaneous level decrease of CNY 42.57 million occurred in expenditure in June 2022. However, the effect on the long-term trend change in expenditure was not statistically significant (*p* = 0.175) (see Figure 1B).

The baseline level of DDDc at start was CNY 11.172. The policy intervention resulted in a statistically significant instantaneous level decrease of CNY 5.427 (*p* < 0.001). Following the intervention, the DDDc exhibited a statistically significant downward trend change of CNY 0.028 per month (*p* < 0.05) (see Figure 1C).

In conclusion, the instantaneous level changes for all different outcome variables were statistically significant upon policy implementation. However, the long-term effect of the NVBP policy was not consistently significant in this study.

### 3.6 ITS analysis of insulin procurement volume for different subgroups

**Figure 2:**
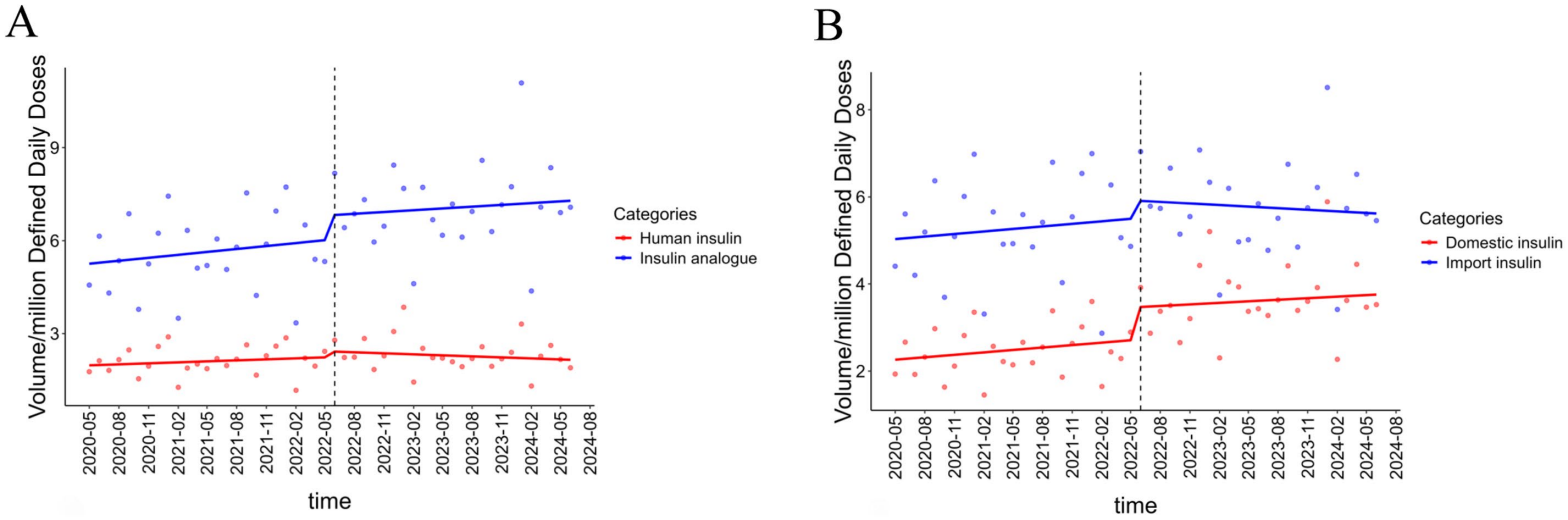
Results of ITSA on insulin procurement volume in different subgroups. **(A)** The result of ITS on volume in human insulin and insulin analogue. **(B)** The result of ITS on volume in domestic insulin and imported insulin.

The results of the ITS analysis on insulin volume across different generations are presented in Table 2. In the month of NVBP implementation, the volume of human insulins increased by 0.439 million DDDs (*p* < 0.001), while that of insulin analogues rose by 1.13 million DDDs (*p* < 0.001). A declining trend in the volume of human insulins and an increasing trend for insulin analogues were observed after NVBP; however, neither trend reached statistical significance (Figure 2A).

ITS analysis of volume by manufacturers is also shown in Table 2. The procurement volume of domestic and imported insulins increased significantly by 0.978 million DDDs (*p* < 0.05) and 0.579 million DDDs (*p* < 0.001) at an instantaneous monthly level, respectively, with a larger increase observed in domestic insulins. A significant upward trend in the volume of domestic insulins was observed following the NVBP policy (*p* = 0.016), whereas imported insulins showed a non-significant downward trend (*p* = 0.342) (Figure 2B).

## 4 Discussion

The present study examined the impact of the NVBP policy on the use of insulin through interrupted time series analysis using the procurement data from Guangdong. Overall, we found that after the implementation of the NVBP policy, the procurement volume of insulin increased in an instantaneous level, while the procurement expenditure and DDDc decreased. And there is a rise in the proportion of high-quality insulin analogue and domestic insulin, which improving overall medication quality level and promoting domestic substitution.

The NVBP policy for insulin has effectively saved expenditure. Compared with the 2 years prior to the procurement, the expenditure on insulin in Guangdong Province decreased by CNY 852.24 million in the 2 years following the procurement. According to the National Health Service Development Statistical Bulletin (30), the total national health expenditure in China surpassed CNY 9 trillion in 2023, accounting for 7.2% of GDP. The growth rate of total health expenditure not only exceeded the GDP growth rate in China but also surpassed the level of OECD countries (31, 32), posing a certain challenge to healthcare insurance fund. The reduction in pharmaceutical expenditure after NVBP has properly played a substantial role in controlling the rapid increase in health expenditure and improved the sustainability of healthcare insurance fund. However, the saving expenditure from the NVBP policy are primarily used to support the development of innovative drugs and the clinical application of new technologies, which may dilute the overall impact on healthcare system costs.

Patients’ accessibility and affordability to insulin significantly improved after NVBP. Insulin procurement volume increased substantially after NVBP, enhancing availability for patients to access affordable insulin, which aligns with findings from Wang et al. (33), Zhao and Wu (26), and Yuan et al. (15, 34), as well as Chen et al. (35) that focused on centralized procurement for chemical drugs. However, long-term analysis revealed no sustained upward trend in volume, which may be attributed to the biphasic insulin (e.g., insulin degludec and insulin aspart) included in medical insurance (33), exerting a alternative effect on winning insulin in NVBP. The research by Chen et al. (36) and Zhang et al. (37) pointed out that there was a negative correlation between economic indicators and insulin use. Due to the high per capita GDP in southern China, the proportion of new hypoglycemic drugs is higher. Therefore, the long-term trend of insulin volume not showing a significant increase may also be related to the rapid increase in the clinical use of new hypoglycemic drugs, such as SGLT-2 inhibitors, GLP-1 receptor agonists, and DPP-4 inhibitors. The result indicates that the impact of NVBP policy is more accurately described as a one-time structural ‘shock’ that reshaped price and market dynamics, exploring a new equilibrium. The market initially responded to the shock, but the absence of a clear, continuous long term trend. Therefore, while the NVBP policy may have delivered short-term benefit in terms of price reduction and efficiency gains, its long term influence on healthcare accessibility and system sustainability remains uncertain (7). This calls for ongoing monitoring and flexible policy adaptation to ensure that any negative longer-term consequences are addressed promptly.

The DDDc of insulin decreased from CNY 11.24 to CNY 5.57 after the policy implementation—a reduction exceeding 50%. The observed reduction in DDDc provides substantial financial relief to many insulin-dependent patients, particularly those who were previously struggling to afford this life-sustaining medication. However, a nearly 50% decrease in DDDc of insulin does not directly indicate that patients with diabetes have reduced the same expenditure, since insulin expenditure is only a part of the expenditure of patients with diabetes, and there may even be a compensatory cost shift phenomenon that other diabetes-related treatment or examination expenses increased after the decrease in insulin price. Therefore, future research can comprehensively evaluate the impact of the NVBP policy on patient expenditure from the perspective of diabetes management.

The NVBP policy has facilitated the upgrading of insulin usage, thereby optimizing the insulin utilization pattern in China. Compared with human insulins, insulin analogues exhibit superior pharmacokinetic properties, more closely mimicking the natural secretion pattern of insulin in human body, which associating with a lower risk of treatment-related hypoglycemic events, improving glycemic control, offering greater flexibility and convenience in injection timing, and effectively enhancing patient medication adherence and treatment outcome (38, 39). The volume of insulin analogues increased more significantly than that of human insulins, with an increased market share after NVBP. This may highlight a difference between the effect of the NVBP policy for biologics and chemical drugs. Centralized procurement of biologics may enhance patient access to upgraded products by narrowing the price gap between normal and upgraded products, thereby promoting the innovation and upgrading of biologics. In Guangdong Province, the average usage proportion of insulin analogues increased from 72.73 to 75.23%, indicating an upgrading of patient medication structure. The volume proportion of insulin analogues exceeded that of some developed countries (40). According to the NHSA, the proportion of insulin analogues use has increased to 70% at the national level following the implementation of centralized procurement, bringing it closer to the medication structure seen in European countries. Following the completion of the insulin procurement agreement in 2024 and subsequent renewal procurement, the national reported volume for human insulin was approximately 76 million shots, while that for insulin analogue insulin was about 165 million shots (41). The proportion of insulin analogue increased from 58% at the initial reporting to 69%, reflecting the optimization of insulin utilization pattern in China. Furthermore, we found a significant portion of the overall reduction in insulin procurement expenditure (91.08%) can be attributed to the reduction in insulin analogues expenditure, consistent with the findings of Yuan et al. (7). After the NVBP, the DDDc difference between human insulins and insulin analugues narrowed, promoting the availability for patients with diabetes to acquire more high-quality insulin analogues.

The reliance on imported insulin has been reduced after NVBP, mitigating supply chain risks. Following NVBP, the proportion of domestic insulin in volume increased from 31.8 to 38.9%, expanding its market share. The procurement volume of domestic insulin has shown a significant upward trend after NVBP, while imported insulin has exhibited a non-significant decline, promoting the substitution of domestic insulin for imported insulin to some extent. Domestic insulin manufacturers get the opportunity for rapid hospital adoption attributing to the NVBP policy, accelerating the domestic substitution process, which is beneficial for reducing supply chain risks and enhancing supply stability under the global tariff uncertainty. Similar findings were reported by Wang et al. (42), who, using a difference-in-differences approach, demonstrated that the NVBP Policy effectively promoted the substitution of original drug by generic drug.

This quantitative analysis of insulin procurement demonstrates that China’s volume-based procurement policy has effectively reduced healthcare expenditure while improving medication accessibility and affordability. The policy has successfully facilitated therapeutic upgrading of insulin products, enhanced supply chain resilience, and embodied the principles of value-based healthcare, offering valuable insights for medical reforms in other countries.

## 5 Limitation

Firstly, this study used data only from Guangdong due to data availability. While Guangdong is a major province economically and demographically in China, with a per capita GDP is equivalent to that of some European and South American countries. The utilization pattern of insulin may be different from that of some economically underdeveloped provinces in China, so it is not suitable for direct extrapolation to the national situation.

Secondly, although this study utilized data from 25 observations both before and after the policy intervention, meeting the requirement for fitting an interrupted time series model, the 25-month post-policy period may still be insufficient to fully assess the long-term effect of the policy. Future research should extend the observation period to further validate the findings of this study.

Finally, the study focused on the impact of the NVBP policy on insulin utilization, without accounting for other potential influencing factors—such as per capita GDP, education levels, or the utilization of new hypoglycemic agents —that could affect observed outcomes. Additionally, the constitution of healthcare costs is a highly complex and interconnected systemic issue, reductions in insulin price do not be consistent to decreased expenditures for patients with diabetes or lower overall healthcare costs. Future research should further investigate potential spillover effects of insulin expenditure saving on other medical expenditure.

## Data Availability

The original contributions presented in the study are included in the article/Supplementary material, further inquiries can be directed to the corresponding author.
