# Supplementary material for: Impact of China’s National Volume-Based Procurement policy exclusively for insulin on the volume, expenditure and price: an interrupted time series analysis in Guangdong Province
Source: Front Public Health. 2025 Sep 8;13:1659721. doi: 10.3389/fpubh.2025.1659721 (PMC12450972; doi:10.3389/fpubh.2025.1659721)
Supplement: Supplementary file 1 [file Table_1.DOCX]

**eTable1. Insulin Volume, Expenditure and price in Guangdong Province, from May 2020 to June 2024**

| **Categories** | | **Before the NVBP (2020.05-2022.05)** | | | | **After the NVBP (2022.06-2024.06)** | | | |
| --- | --- | --- | --- | --- | --- | --- | --- | --- | --- |
|  |  | Procurement Amount/million shots | Volume/million DDDs | Expenditure/million CNY | DDDc/CNY | Procurement Amount/million shots | Volume/million DDDs | Expenditure/million CNY | DDDc/CNY |
| **Total** | | 25.00 | 192.40 | 2165.90 | 11.24 | 29.71 | 235.80 | 1313.66 | 5.57 |
| **Generations** | Human insulin | 6.36 | 52.47 | 267.95 | 5.11 | 6.94 | 58.41 | 191.91 | 3.29 |
|  | Insulin analogue | 18.64 | 139.92 | 1897.95 | 13.53 | 22.78 | 177.39 | 1121.75 | 6.32 |
| **Enterprises** | Domestic insulin | 7.56 | 61.27 | 487.76 | 7.94 | 11.38 | 91.67 | 387.12 | 4.23 |
|  | Imported insulin | 17.44 | 131.13 | 1678.14 | 12.76 | 18.33 | 144.13 | 926.55 | 6.42 |
| **Bidding groups** | Basal human | 0.11 | 0.83 | 4.81 | 5.85 | 0.17 | 1.34 | 5.20 | 3.88 |
|  | Mealtime human | 2.17 | 21.00 | 75.78 | 3.61 | 2.82 | 27.48 | 76.39 | 2.78 |
|  | Premixed human | 4.09 | 30.65 | 187.36 | 6.12 | 3.95 | 29.60 | 110.32 | 3.73 |
|  | Long-acting basal analogue | 6.13 | 46.10 | 1027.74 | 22.29 | 7.99 | 66.48 | 596.93 | 8.98 |
|  | Rapid-acting analogue | 5.03 | 37.72 | 355.94 | 9.44 | 7.03 | 52.75 | 260.83 | 4.95 |
|  | Premixed analogue | 7.48 | 56.10 | 514.27 | 9.16 | 7.75 | 58.15 | 263.98 | 4.53 |

The volume counted in DDDs is the standardized form of the procurement amount counted in shot, therefore, only the volume counted in DDDs is presented in the manuscript.

The insulin was categorized into six bidding groups in process of procurement, including mealtime human insulin, basal human insulin, premixed human insulin , rapid-acting analogue (aspart, lispro, glulisine), long-acting basal analogue (glargine, detemir, degludec) and premixed analogue (protamine lispro and lispro, protamine aspart and aspart).

**eTable2. Results of ITS Analysis for Insulin Volume, Expenditure, and DDDc**

| **Categories** | **Baseline level** | **Baseline trend** | **Level change** | **Trend change** |
| --- | --- | --- | --- | --- |
| **Volume/million DDDs** |  | | | |
| Overall | 7.32^***^ | 0.007(0.612) | 1.547^***^ | 0.008(0.629) |
| Human insulin | 2.039^***^ | -0.002(0.616) | 0.439^***^ | -0.009(0.080) |
| Insulin analogue | 5.299^***^ | 0.009(0.292) | 1.13^***^ | 0.017(0.149) |
| Domestic insulin | 2.296^***^ | 0.003(0.392) | 0.978^***^ | 0.016^**^ |
| Imported insulin | 5.04^***^ | 0.006(0.477) | 0.579^***^ | -0.009(0.346) |
| Basal human | 0.029^***^ | 0(0.498) | 0.029^***^ | -0.001^*^ |
| Mealtime human | 0.726^***^ | 0.007^***^ | 0.203^***^ | -0.007^***^ |
| Premixed human | 1.249^***^ | -0.004(0.606) | 0.235(0.140) | -0.012(0.275) |
| Long-acting basal analogue | 1.581^***^ | 0.015^**^ | 0.35^**^ | 0.008(0.220) |
| Rapid-acting analogue | 1.338^***^ | 0.011^***^ | 0.214^***^ | 0.008^***^ |
| Premixed analogue | 2.246^***^ | -0.006^*^ | 0.285^***^ | -0.001(0.610) |
| **Expenditure/million CNY** |  | | | |
| Overall | 81.18^***^ | 0.71(0.078) | -42.57^***^ | -0.722(0.175) |
| Human insulin | 10.764^***^ | 0.022(0.290) | -2.038^***^ | -0.101^***^ |
| Insulin analogue | 68.724^***^ | 0.702^***^ | -38.071^***^ | -0.747^***^ |
| Domestic insulin | 18.08^***^ | 0.167(0.157) | -7.365^**^ | -0.064(0.682) |
| Imported insulin | 63.099^***^ | 0.543(0.068) | -35.205^***^ | -0.658(0.095) |
| Basal human | 0.19^***^ | 0.001(0.669) | 0.045(0.141) | -0.004(0.100) |
| Mealtime human | 2.72^***^ | 0.033^***^ | -0.368^***^ | -0.032^***^ |
| Premixed human | 7.931^***^ | -0.014(0.319) | -2.24^***^ | -0.04^**^ |
| Long-acting basal analogue | 34.906^***^ | 0.526^***^ | -23.704^***^ | -0.426^***^ |
| Rapid-acting analogue | 12.506^***^ | 0.161^***^ | -6.29^***^ | -0.097^***^ |
| Premixed analogue | 20.446^***^ | 0.067^*^ | -9.017^***^ | -0.2^***^ |
| **DDDc/CNY** |  | | | |
| Overall | 11.172^***^ | 0.014(0.333) | -5.427^***^ | -0.042^*^ |
| Human insulin | 5.286^***^ | -0.012^*^ | -1.49^***^ | -0.002(0.683) |
| Insulin analogue | 13.456^***^ | 0.016(0.385) | -6.754^***^ | -0.06^*^ |
| Domestic insulin | 7.928^***^ | 0.007(0.649) | -3.674^***^ | -0.015(0.445) |
| Imported insulin | 12.624^***^ | 0.02(0.212) | -6.067^***^ | -0.056^*^ |
| Basal human | 6.249^***^ | -0.03^***^ | -1.64^***^ | 0.033^***^ |
| Mealtime human | 3.715^***^ | -0.007^**^ | -0.668^***^ | 0.001(0.840) |
| Premixed human | 6.225^***^ | -0.008^*^ | -2.269^***^ | 0.006(0.257) |
| Long-acting basal analogue | 22.435^***^ | 0.007(0.827) | -12.219^***^ | -0.088^*^ |
| Rapid-acting analogue | 9.522^***^ | -0.001(0.915) | -3.97^***^ | -0.033^*^ |
| Premixed analogue | 9.245^***^ | -0.001(0.935) | -4.045^***^ | -0.039^*^ |

*P* values corresponding to coefficients are reported in parentheses; * *P* < 0.05, ** *P* < 0.01, and *** *P* < 0.001.
